# InterMine: extensive web services for modern biology

**DOI:** 10.1093/nar/gku301

**Published:** 2014-04-21

**Authors:** Alex Kalderimis, Rachel Lyne, Daniela Butano, Sergio Contrino, Mike Lyne, Joshua Heimbach, Fengyuan Hu, Richard Smith, Radek Štěpán, Julie Sullivan, Gos Micklem

**Affiliations:** Department of Genetics, University of Cambridge, Downing Street, Cambridge, CB2 3EH, UK and Cambridge Systems Biology Centre, University of Cambridge, Tennis Court Road, Cambridge, CB2 1QR, UK

## Abstract

InterMine (www.intermine.org) is a biological data warehousing system providing extensive automatically generated and configurable RESTful web services that underpin the web interface and can be re-used in many other applications: to find and filter data; export it in a flexible and structured way; to upload, use, manipulate and analyze lists; to provide services for flexible retrieval of sequence segments, and for other statistical and analysis tools. Here we describe these features and discuss how they can be used separately or in combinations to support integrative and comparative analysis.

## INTRODUCTION

InterMine is an open-source data warehouse system used for creating large biological databases of heterogenous data sources with fast, customizable query facilities (1). The model-driven system enables rapid extension to new data types, and essentially automatic denormalization enables high performance, as well as tuning of live databases. Originally developed for the FlyMine project (2), a database of integrated *Drosophila* genomic and proteomic data, InterMine now underpins several projects around the world (3,4,5,6,7,8) including warehouses at many of the leading model organism databases (MODs) (Table 1). The MOD-InterMine databases serve as advanced query interfaces for the MODs, and include rich MOD-curated data together with many datasets from external sources, for instance protein interaction data and ortholog gene sets.

**Table 1.** Publicly available InterMine databases

Database	Web service URI
FlyMine	http://flymine.org/query
MouseMine	http://mousemine.org/mousemine
RatMine	http://ratmine.mcw.edu/ratmine
YeastMine	http://yeastmine.yeastgenome.org/yeastmine
WormMine	http://intermine.wormbase.org/tools/wormmine
ZebrafishMine	http://zebrafishmine.org
modMine	http://modmine.org/query
metabolicMine	http://metabolicmine.org/beta
MitoMiner	http://mitominer.mrc-mbu.cam.ac.uk/release-x.x
FlyTF	http://flytf.org/query
TargetMine	http://targetmine.nibio.go.jp/targetmine

**Figure 1.**
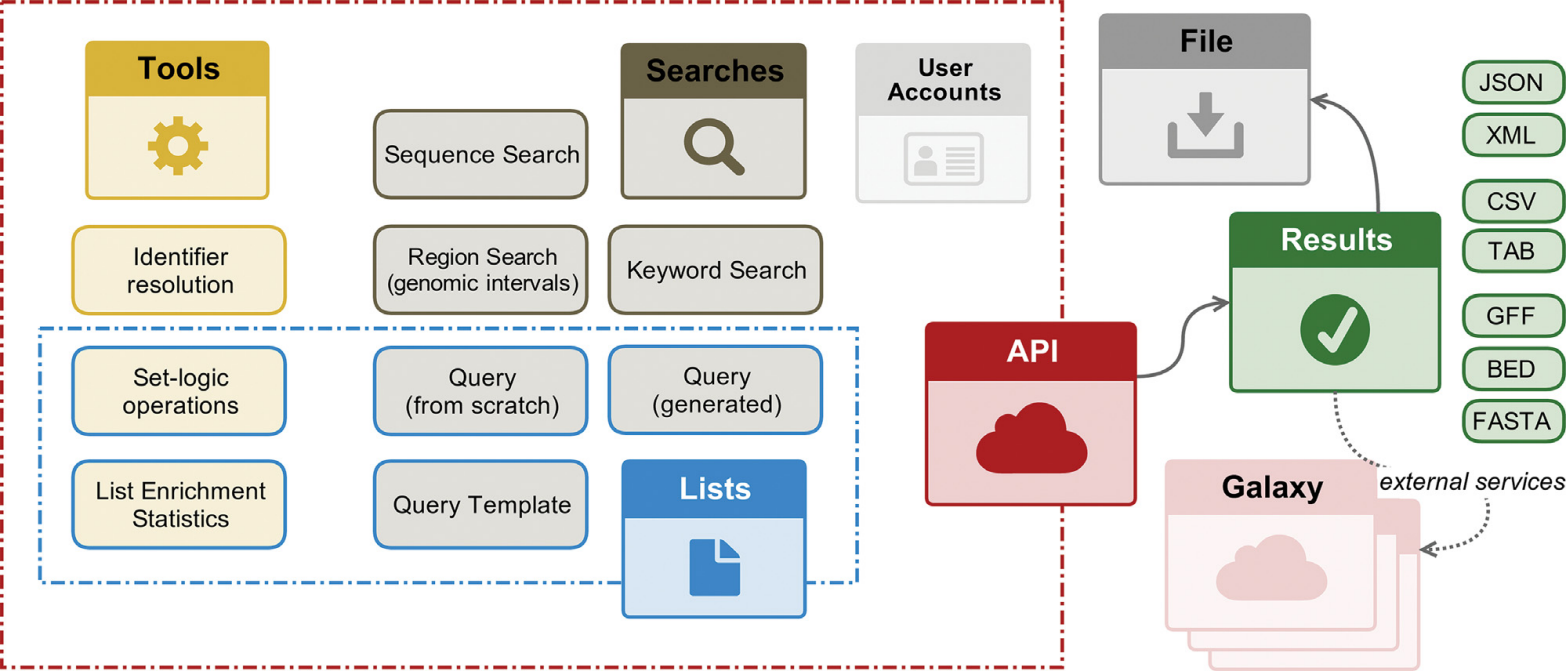
Summary of web services available through InterMine.

InterMine as a platform provides a customizable web interface in which users can run searches interactively using either pre-defined search forms (templates) or build their own query using a query builder. Searches can be run for single entities such as a gene, or sets of entities such as a gene list. In addition, analysis tools are provided, such as gene set enrichment and set operations. Complementary to the web interface, all of the search and analysis functions are available via web services (Figure 1). These expose core functionality (queries, templates and lists) as well as metadata (such as the data model) and specialized resources (such as DNA sequence export, region search and enrichment statistics). The web services enable users to automate data-based workflows or access data directly without using the InterMine web application. In addition, the presence of a common web service API means that it is easier to write programs that interrogate more than one InterMine database, thus allowing users to draw together data from disparate sources. Due to their modular nature, the web services also enable specific functions to be used as stand alone components within third party web interfaces. For example, the data navigation for the modENCODE website (www.modencode.org) is generated by web services from modMine. In addition, SGD has developed an app for the iPhone, iPad and iPod touch called ‘YeastGenome’, which is powered by the InterMine web services and provides access to the latest *Saccharomyces cerevisiae* information from YeastMine (www.yeastgenome.org/yeastgenome-app-information). The web services are also used to provide seamless data upload from InterMine to external analysis suites including Galaxy (http://galaxyproject.org/) and Genomespace (http://www.genomespace.org/).

A full description of the InterMine system was published at the end of 2012 (1). Since then, most work has focused on improvements to the web interface including a feature for sharing lists between users. However, of relevance to the web services have been improvements to the enrichment widgets that now allow the users to select their own background population and normalize by gene length, and the addition of a sequence extraction service where the sequence of any defined chromosomal region can be returned, a feature not yet available in the web interface. These features are described in more detail below. InterMine is an active project with many new features planned, including more powerful search functions and data visualization.

### The web services

InterMine's web services make use of standard HTTP 1.0 method calls (often termed a RESTful web service) between client and server and thus the HTTP request methods GET and POST can be used to send and receive queries and data using a command line program such as Curl (http://curl.haxx.se/). While any language capable of making standard HTTP requests can be used, client library support is provided in five commonly used programming languages—Python, Perl, Java, JavaScript and Ruby. The web services enable users to build and execute searches and fetch data in a number of formats (such as JSON, XML, tabular, GFF3 and FASTA).

Throughout the paper (and Supplementary Materials), we will introduce web service URI examples for illustration that can be tested in the browser or retrieved using an appropriate command line program. These are not meant to be human readable but are intended to be parsed programmatically. The individual client libraries are able to translate results into human readable formats.

A web service call URL consists of a base, which defines the InterMine instance, the resource path that specifies the functionality required, the service method and any additional parameters. For each InterMine instance, a full list of available web services can be accessed using the /service endpoint with results returned in JSON format (available from version 14 of the API onwards). For example, a list of available web services for FlyMine can be retrieved as follows:

GET ‘http://www.flymine.org/query/service’

The service listing is consumed by IOdocs (http://iodocs.labs.intermine.org), which provides InterMine's API documentation for the web service resources, their methods and parameters (see the Supplementary Materials for more details). Here, resources are automatically exposed as executable examples, meaning that, for each method, a user can review and edit input parameters and, in the browser, see the result of running the query.

### Data search and retrieval

Querying the data via web services works similarly to searching the data via the InterMine web application. The keyword search is a free text, faceted search, meaning that searches not only return items of interest but also give a breakdown of the matching classes/categories, and how many matches each contains. InterMine also offers a structured query that allows for a more finely tuned search to be run: users can build their own query from scratch using the client libraries or use one of the pre-built template queries.

### Keyword search

A simple keyword search can be performed by issuing a ‘GET’ method to the /search resource; here supplying a *Drosophila* gene symbol—‘eve’:

GET ‘http://www.flymine.org/query/service/search?q=eve’

This searches the entire database for the keyword ‘eve’ and returns all relevant results, along with a score for each record, with a higher score indicating a better match (see listing A). The keyword search supports wildcards and boolean search syntax so search phrases like ‘dros*’ and ‘fly AND NOT embryo’ are valid. The keyword search is not case sensitive.

**Listing A:**
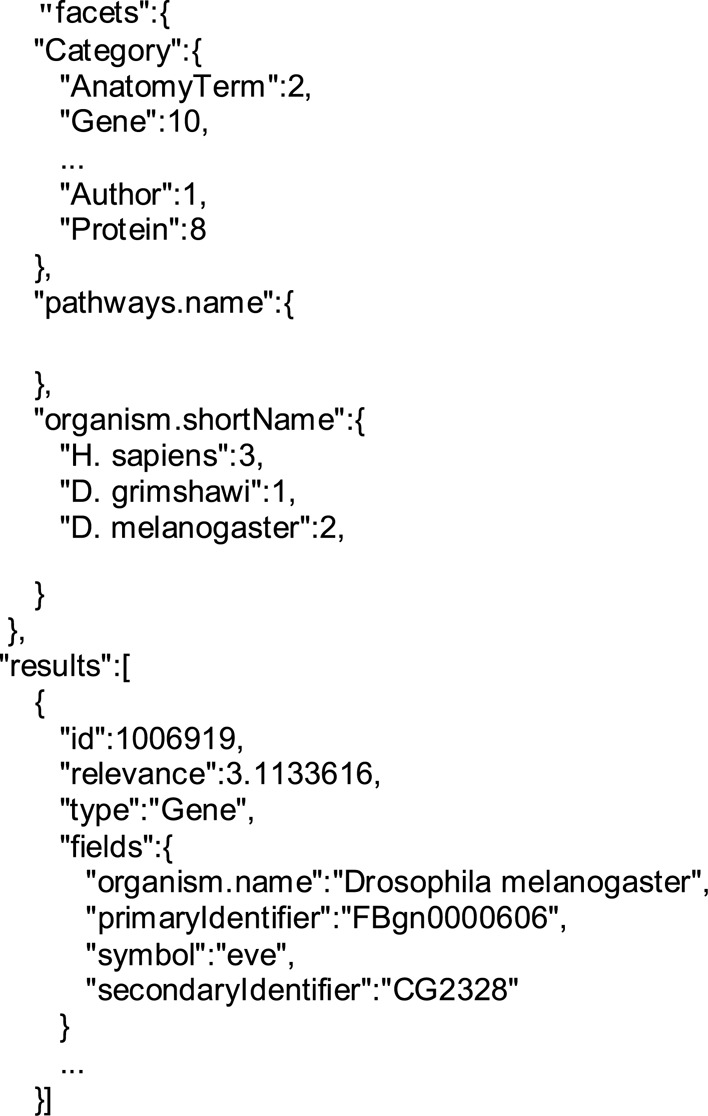
Truncated results in JSON format when searching for the keyword ‘eve’. The header of the results of a keyword search includes the facets. ‘Category’ is the summary of the types of data returned and, for each, the number of matching records. In this specific search, no pathways were found so that section is empty. The last facet lists the relevant organisms found.

The first record will be the facets for the search just run; facets are attributes of the search results and can be added as parameters to filter the search results, for instance adding ‘facet_Category = Gene’ will filter the search results to only include genes:

GET ‘http://www.flymine.org/query/service/search?q=eve&facet_Category=Gene’

### Template queries

Additionally, the curators of each InterMine database provide a library of *Templates*: web services and corresponding search forms with a set of pre-defined constraints. These templates represent common searches and tasks often generated in response to user requests. For example, a template search to retrieve Gene Ontology (GO) annotations for a particular gene is available in a number of the InterMine databases (including FlyMine, YeastMine, ZebrafishMine, RatMine and MouseMine) and can be run on a single gene or many genes that have either been previously stored as a list in the relevant InterMine or automatically created as part of a script. Public template searches are constructed using the *query builder* (see below) by super-users, while ordinary users can construct templates for their own use. A list of the templates available in an InterMine can be retrieved with the ‘/query/templates’ method or by viewing the templates page in the webapp, e.g. http://yeastmine.yeastgenome.org/yeastmine/templates.do.

### Client libraries

The InterMine project publishes software ‘client’ libraries in a variety of common programming languages that handle common tasks, such as correctly formatting parameters, generating valid query XML, interpreting the data model, parsing results and making basic HTTP requests. This enables users to focus on the problem they are trying to solve, rather than the mechanism of using an HTTP-based API.

Automatically generated code for the client libraries can be accessed from each template form on the web interface, where there are links to (i) the web service URL providing a permanent link to run the template again from a script or browser, (ii) ready-to-use code for each of the client languages and (iii) generated XML representing the query (which can be modified and used in HTTP requests) can be found. In addition, the InterMine web interface includes a *query builder* that allows the editing of templates and the construction of custom queries based on InterMine's data model. This interface (and any results table produced from it) can also export the query itself, either serialized in XML or as part of a generated program using one of the supported client libraries. Such automatically generated code helps users start exploiting the client libraries. See the Supplementary Material for more detail on accessing and using the client libraries.

### Search results

The results of a search can be returned in several generic formats, including JSON, XML and tabular (tab or comma-delimited), allowing storage or further processing. In addition specific modules provide access to biology-specific formats including GFF3, FASTA and BED formats.

### Sequence and chromosome interval searches

The sequence of any defined chromosomal region can be returned through the web services. Here, the web service extends the sequence retrieval capabilities of the web interface, where only the sequence of defined features mapped to a chromosome can be returned. Using the web services the user has the added benefit of being able to return the sequence of any feature extended upstream or downstream by a specified amount (see the Supplementary Material, Section 3b, for more details).

Chromosome intervals constitute the output of many different types of biological experiments and may contain, for example, binding sites, polymorphisms or genes of interest. InterMine provides simple methods to search such intervals for features of biological relevance. This functionality is part of the Path-Query API and enables a search for features covering a range of types (for example, SNPs, genes, exons and binding sites) that overlap a specified set of intervals in a given organism. The feature results may either be retrieved as a normal query result, in sequence annotation formats such as GFF3 and BED, or stored as a list of features on the originating server. For example, the code shown in listing B illustrates a search for exons in a region of *D. melanogaster* chromosome 2L, processing the results as standard query results.

**Listing B:**
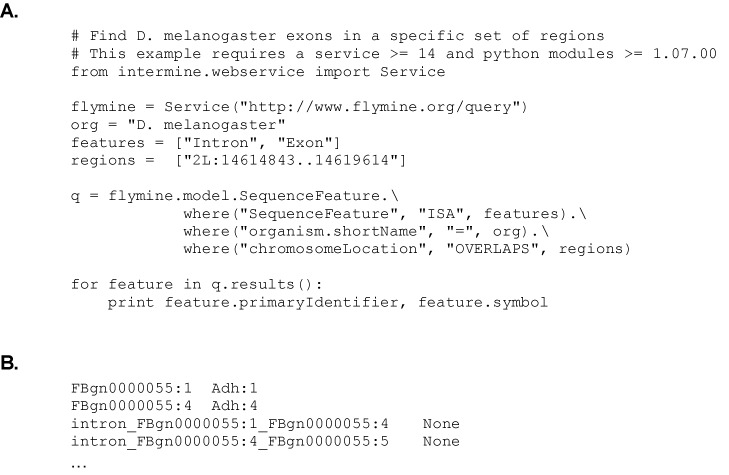
**A.** Python code illustrating how a search can be made for features (in this case Introns and Exons) mapped to a defined chromosomal region (in this case Chromosome 2L: 14614843..14619614 from FlyMine). **B.** Output from the python code (full results are not shown) showing two exons (FBgn0000055:1 Adh:1 and FBgn0000055:4 Adh:4) and two introns from the Adh gene.

### Lists and list analysis tools

In InterMine, users can work with sets of objects, called ‘lists’. Commonly created by list upload or generated from search and analysis results, lists can be used in web service requests, as input in template searches and custom queries, derived from other sets using set-logic operations and analyzed with analysis widgets. Web service methods are available for all aspects of list management, including modifying list contents, deleting lists, list union and subtraction, and sharing lists with other users.

The InterMine system provides a method of *identifier resolution* for creating lists of objects from sets of identifiers that may include old or heterogenous members. This method allows users to assess which matches are suitable for their lists, either on an individual basis or categorized by resolution method (identified duplication, use of out-dated synonym, matching multiple items, etc.). See the Supplementary Material, Section 3a, for some example code.

Since the creation of a list requires storing information permanently on an InterMine server, saving of new lists and access to lists stored within the user's InterMine account require authentication using a supplied API access token (accessible under the ‘Account Details’ tab in the user's MyMine account).

InterMine includes a growing number of analysis tools including gene set enrichment widgets and set-logic operations. The web services expose the functionality of all these tools. For example, list set operations allow union, intersection, symmetric difference and subtraction to be performed on lists.

The gene set enrichment tool looks for annotations to a set of genes that occur more than would be expected by chance, given a background population of annotations. The hypergeometric distribution is used to calculate a *P*-value for each annotation and a choice of correction methods for multiple testing (Bonferonni, Holm-Bonferonni and Benjamini Hochberg) are available. The calculation of such enrichment statistics for gene set analysis is now commonplace, particularly for the analysis of GO terms annotated to a set of genes (9). InterMine provides GO enrichment statistics as well as enrichment statistics for other annotation types including protein domains, pathways, human diseases, mammalian phenotypes and publications. The default background population is all genes in the genome with the specified annotation type. However, the background population can be changed by specifying another list. Output from enrichment for human diseases (MEDIC ontology terms, 10) for a list of 321 mouse DNA repair genes is shown in listing C.

**Listing C:**
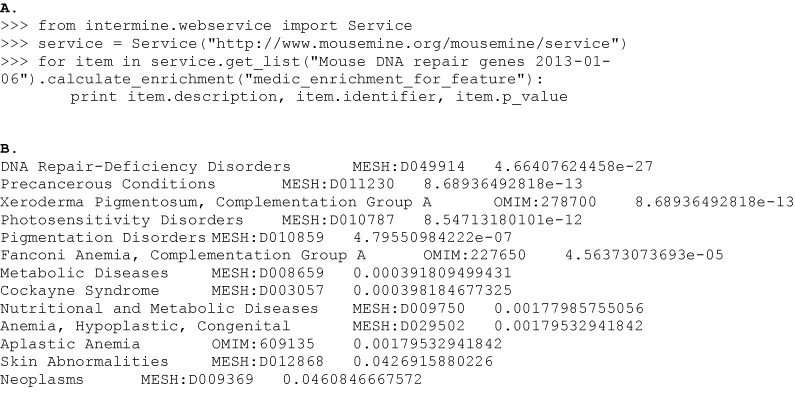
**A**. Python code calculating enrichment statistics for MEDIC (10) ontology terms on a public list of 321 mouse DNA repair genes. The full gene list (Mouse DNA repair genes 6 January 2013) can be viewed at http://www.mousemine.org/mousemine/bag.do?subtab=view. **B**. The output from the code in (**A**): MEDIC is a merging of OMIM disease identifiers onto the MeSH disease ontology, thus the output includes MeSH and OMIM terms. Each line displays the MeSH/OMIM ontology term, the ontology ID and the calculated P-value.

## DISCUSSION

InterMine's core functionality is driven through comprehensive web services; these are used to build the web interface and provide result sets, list functionality and user/group management. The web services enable automation of comparative analysis of data across all existing InterMine instances. In particular, this allows creation of reports that draw on data from more than one InterMine database and researchers can write their own analysis pipelines utilizing any or all of the databases allowing for automation of time-consuming or repetitive tasks. The web services enable all the functionality otherwise provided by the web interface, including searches, results and tools, to be seamlessly joined in a pipeline. As a simple example, the query outlined above in listing C, in which a list of mouse genes is shown to be enriched for DNA repair MEDIC terms, could be extended to return human genes annotated with the same terms. This approach combines web services for list use, list enrichment tools and template searching (the pipeline is described in more detail in the Supplementary Materials, Section 3c, together with further example usage of the web services).

The InterMOD project (11) is building on the ideas presented above and working towards unification of parts of the data model of the MOD-InterMine databases, thus making such cross-organism analyses more straightforward. Providing a common web services API to access the different InterMine instances brings opportunities to combine disparate data from multiple organisms in a standard format. Similarly, the other web services described in this issue provide opportunities for powerful integrative analysis in combination with the services and data available through InterMine warehouses.

InterMine is an active project with a worldwide team of developers and users, and core funding currently in place until 2018. Regular releases with new features and tools are made. All our code is hosted on Github (http://github.com/intermine/) and contributions are welcome. All InterMine code is free and open-source and released under the Lesser GNU Public License (http://www.gnu.org/licenses/lgpl.html). Extensive documentation is available for the web services at http://intermine.readthedocs.org/en/latest/web-services/ and further details are available in the Supplementary Material, Section 1.

## SUPPLEMENTARY DATA

Supplementary Data are available at NAR Online.

Supplementary Data
