# Supplementary material for: InterMine: extensive web services for modern biology
Source: Nucleic Acids Res. 2014 Apr 21;42(Web Server issue):W468–72. doi: 10.1093/nar/gku301 (PMC4086141; doi:10.1093/nar/gku301)
Supplement: Supplementary Data [file supp_42_W1_W468__index.html]

Supplementary Data 

# InterMine: extensive web services for modern biology

## Supplementary Data

**Files in this Data Supplement:**

- SUPPLEMENTARY DATA
