# Supplementary material for: InterMine: extensive web services for modern biology
Source: Nucleic Acids Res. 2014 Apr 21;42(Web Server issue):W468–72. doi: 10.1093/nar/gku301 (PMC4086141; doi:10.1093/nar/gku301)
Supplement: Supplementary Data [file supp_gku301_nar-00255-web-b-2014-File003.pdf]

InterMine: Extensive Web Services for Modern Biology - Supplementary Material

Contents

1. Documentation
    a. API documentation
    b. Cookbooks
    c. Interactive documentation
2. Getting Started - using the client libraries
3. Code Examples
    a. Identifier resolution in list upload
    b. Retrieving a sub-sequence
    c. Building analysis pipelines

1. Documentation

a. API Documentation

Documentation is available which describes InterMine's web service resources, their methods and parameters (table S1), all of which can also be accessed from the web service section of the InterMine documentation: http://intermine.readthedocs.org/en/latest/web-services. In addition, most InterMine instances have a link to the API documentation on the home page and a navigation tab at the top dedicated to web services (figure S1). If you have trouble, each InterMine has a contact form at the bottom of each page which sends an email to the relevant help desk.

	**Download**	**Docs**
**HTTP API**	http://iodocs.labs.intermine.org	
**Java Client**	http://lib.intermine.org/java	http://ci.intermine.org/job/docs/javadoc/
**Perl Client**	http://search.cpan.org/perldoc?Webservice%3A%3AInterMine	
**Python Client**	http://pypi.python.org/pypi/intermine	http://packages.python.org/intermine/
**Ruby Client**	http://www.rubygems.org/gems/intermine	http://www.rubygems.org/gems/intermine
**JavaScript Client**	https://npmjs.org/package/imjs	http://intermine.github.io/imjs

Table S1: Documentation available for the InterMine API and client libraries.

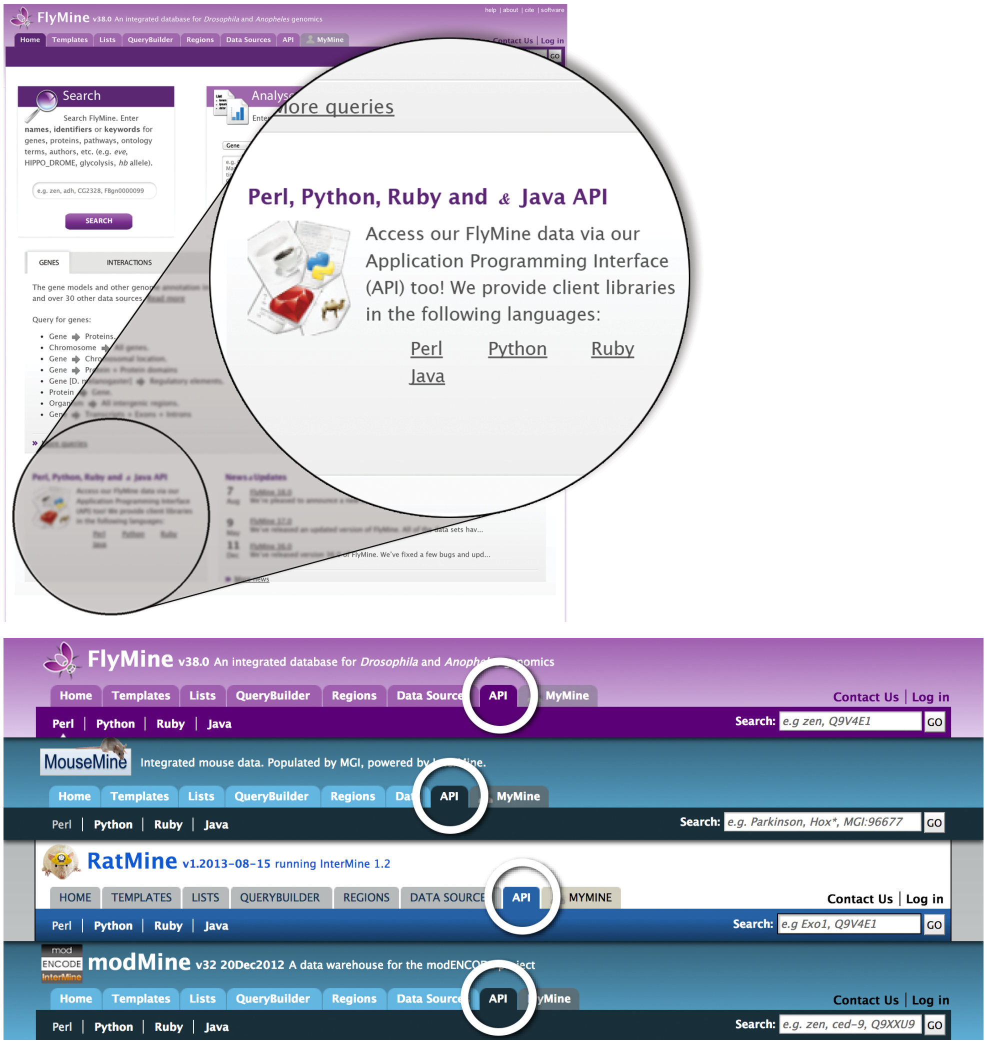

Figure S1: Examples of the links to the API documentation that are available from the home page of each InterMine database and through the main navigation tab of the web interface.

b. Cookbooks

The web services section of the InterMine documentation also has several example scripts:

http://intermine.readthedocs.org/en/latest/web-services/how-do-i/

These “how-tos” detail how to accomplish a specific task, e.g. “How Do I Get a Summary of a Gene?”, using each library available. Users can request new “how-tos” by adding a support ticket.

c. Interactive documentation

For each InterMine instance, a full list of available web services can be accessed using the `/service` endpoint with results returned in JSON format.

The same service listing is consumed by IOdocs which provides InterMine’s API documentation (http://iodocs.labs.intermine.org). Here, resources are automatically exposed as executable examples, meaning that for each method a user can review and edit input parameters and, in the browser, see the result of running the query.

2. Getting started: Using the client libraries

Each template search, custom query (using the query builder) or results table in the web interface includes a mechanism for generating code using one of the client libraries. The code will generate the same results as those generated by the search or as seen in the results table (figure S2). The generated code can help get you started using the web service client libraries. Working from the generated stub, you can edit the code to perform your intended task. You will probably want to refer to the API documentation for your target language (http://intermine.readthedocs.org/en/latest/web-services/#api-and-client-libraries).

To access code from a template search, some familiarity with the web interface is required. All InterMine databases have a similar web interface, hence reducing the learning curve. Each InterMine web page includes a series of tabs allowing navigation between the search and tool functions. One such tab, ‘Templates’, provides access to a library of template queries for that InterMine instance. Templates are simple forms with drop-down lists and text boxes with auto-completion, where users can specify filters. The code generated from a template form will reflect any user constraints added to the form (see figure S2).

Figure S2 illustrates code accessed from a template search for all GO annotations for a specified gene. A similar search could be constructed in the query builder, or indeed displayed directly in the query builder from the template form (by clicking the ‘Edit query’ button). Like the templates, the query builder can also be accessed through the navigation tabs in the web interface. Automatically generated code can be accessed from within the query builder itself or from the results table produced by running the query. The following python code was generated from such a query in FlyMine:

```
# Get a new query on the class (table) you will be querying:
query = service.new_query("Gene")

# The view specifies the output columns
query.add_view(
    "symbol", "secondaryIdentifier", "goAnnotation.ontologyTerm.identifier",
    "goAnnotation.ontologyTerm.name", "goAnnotation.evidence.code.code",
    "goAnnotation.ontologyTerm.namespace", "goAnnotation.qualifier"
)

# This query's custom sort order is specified below:
query.add_sort_order("Gene.secondaryIdentifier", "ASC")

# You can edit the constraint values below
query.add_constraint("Gene", "LOOKUP", "CG11348", "", "A")

# Uncomment and edit the code below to specify your own custom logic:
# query.set_logic("A")

for row in query.rows():
    print row["symbol"], row["secondaryIdentifier"],
row["goAnnotation.ontologyTerm.identifier"],
        row["goAnnotation.ontologyTerm.name"],
row["goAnnotation.evidence.code.code"],
        row["goAnnotation.ontologyTerm.namespace"],
row["goAnnotation.qualifier"]
```

To run the code samples it is assumed that you have followed the online documentation and have both the programming environment and the appropriate webservice client library module installed. The Python client library used in the examples has been tested on Python 2.5, 2.6 & 2.7, on Linux, Mac OS X and Windows operating systems, and like all our code is open-source, licensed under the LGPL. Extensive documentation is available detailing the installation and use of each of the client libraries: http://intermine.readthedocs.org/en/latest/web-services/#api-and-client-libraries

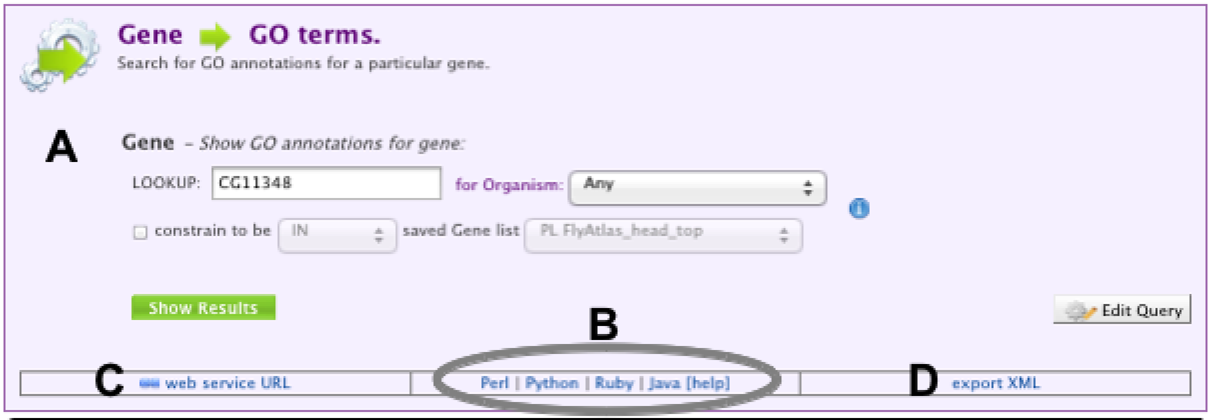

Perl

```
# The following import statement sets FlyMine as your default
use Webservice::InterMine 0.9904 'http://www.flymine.org/release-
38.0';
# Description: Search for GO annotations for a particular gene.
my $template = Webservice::InterMine->template('Gene_GO')
    or die 'Could not find a template called Gene_GO';
# Use an iterator to avoid having all rows in memory at once.
my $it = $template->results_iterator_with(
    # A:  Gene - Show GO annotations for gene:
    opA    => 'LOOKUP',
    valueA => 'CG11348',
);
while (my $row = <$it>) {
    print $row->{'symbol'}, $row->{'secondaryIdentifier'},
        $row->{'goAnnotation.ontologyTerm.identifier'}, $row-
>{'goAnnotation.ontologyTerm.name'},
        $row->{'goAnnotation.evidence.code.code'}, $row-
>{'goAnnotation.ontologyTerm.namespace'},
        $row->{'goAnnotation.qualifier'}, "\n";
}
```

Python

```
# The following two lines will be needed in every python script:
from intermine.webservice import Service
service = Service("http://www.flymine.org/release-38.0/service")
# Search for GO annotations for a particular gene.
template = service.get_template('Gene_GO')
# You can edit the constraint values below
# A    Gene    Show GO annotations for gene:
rows = template.rows(
    A = {"op": "LOOKUP", "value": "CG11348", "extra_value": ""}
)
for row in rows:
    print row["symbol"], row["secondaryIdentifier"],
row["goAnnotation.ontologyTerm.identifier"],
        row["goAnnotation.ontologyTerm.name"],
row["goAnnotation.evidence.code.code"],
        row["goAnnotation.ontologyTerm.namespace"],
row["goAnnotation.qualifier"]
```

Ruby

```
# The following two lines will be needed in every script:
require "rubygems"
require "intermine/service"
service = Service.new("http://www.flymine.org/release-38.0")
# query description - Search for GO annotations for a particular gene.
# Get a new query from the service you will be querying:
service.new_query("Gene").
    select(["symbol", "secondaryIdentifier", "goAnnotation.ontologyTerm.identifier",
"goAnnotation.ontologyTerm.name", "goAnnotation.evidence.code.code",
"goAnnotation.ontologyTerm.namespace", "goAnnotation.qualifier"]).
    # You can edit the constraint values below
    where("Gene" => {:lookup => "CG11348"}).
    limit(10).
    each_row { |r| puts r}
```

Figure S2: **A**. The template search ‘Gene → GO term’ from FlyMine, which returns all GO annotations for a gene or specified list of genes. **B**. The links to generate the client library code that would give the same results as the template search in **A**. The perl, Python and Ruby code generated from this template is shown **C**. The link to generate a web service URL which can be used to fetch results for this template from the command line or a script. For this template the URL is:
http://www.flymine.org/release-38.0/service/template/results?name=Gene_GO&constraint1=Gene&op1=LOOKUP&value1=CG11348&extra1=&format=tab&size=10.
D. The link to generate the query xml for this template. Such XML can be used in HTTP requests.

3. Code Examples

In this section we provide example code for using the web services for both common biological usage - using the identifier resolution in list upload and retrieving sequence from a specified biological region, and in two example pipelines - one producing a suggest service that identifies similar genes by their GO annotation and another performing an enrichment analysis for disease terms on a set of mouse genes, followed by a search for human genes annotated with an enriched term and another

a. Identifier resolution in list upload

```
from intermine.webservice import Service
from datetime import datetime

USAGE = "Usage: python %s uri token data-type file-or-ids [list-name]"

def upload_list(uri, token, data_type, identifiers, name = None):

    # Get a connection to the server
    service = Service(uri, token = token)

    # Construct a suitable name if none was provided
    if name is None:
        name = "New %s list (%s)" % (data_type, datetime.now())

    # Create the list.
    new_list = service.create_list(identifiers, data_type, name = name)

    # Report the list.
    print "Created list", new_list
```

```
    if len(new_list.unmatched_identifiers):
        print "The following identifiers were rejected:"
        for ui in new_list.unmatched_identifiers:
            print " * ", ui

if __name__ == "__main__":
    import sys
    if len(sys.argv) != 5: sys.exit(USAGE % sys.argv[0])
    upload_list(*sys.argv[1:])
```

b. Retrieving a sub-sequence

The ‘sequence’ endpoint (`service/sequence`), may be used to retrieve a sequence object or fetch an indexed sub-sequence of it e.g. a chromosome sub-sequence interval.

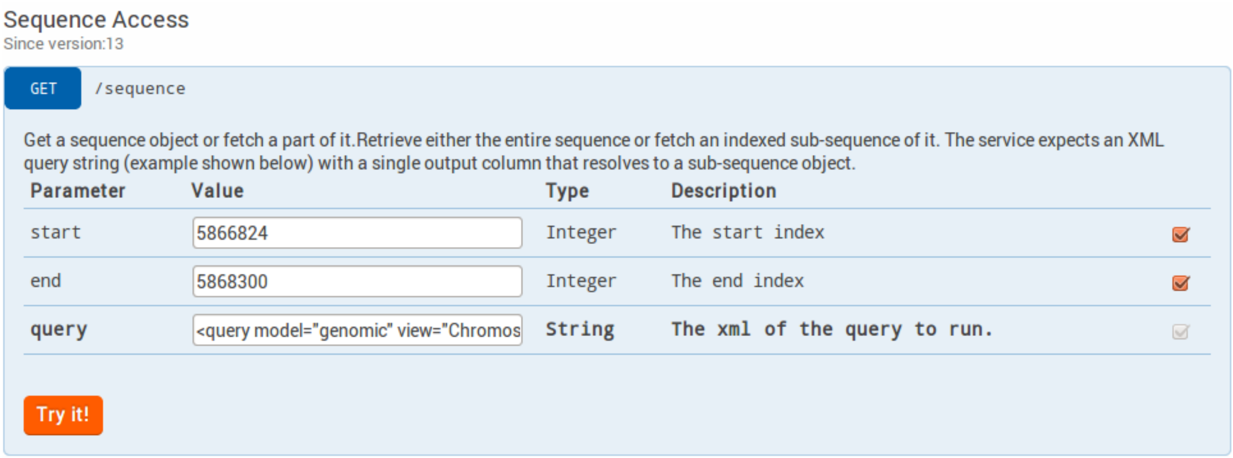

Figure S3. The /sequence method exposed in iodocs

The service expects an XML query string (see figure S3) with a single output column that resolves to a sub-sequence object. Optionally, the user may provide sub-sequence coordinates defining both fragment start and end (integer).

The sequence query uses the ‘genomic’ data model and needs to define the data class - here ‘Chromosome’. Additionally, the query is constrained to `LOOKUP` a particular chromosome identifier and, where necessary, an organism:

```
<query model="genomic" view="Chromosome.sequence.residues">
<constraint path="Chromosome" op="LOOKUP" value="2R" extraValue="D.
melanogaster"/>
```

```
</query>
```

The final URL would look like the example below:

http://beta.flymine.org/beta/service/sequence?start=5866824&end=5868300&query=<query model="genomic" view="Chromosome.sequence.residues"><constraint path="Chromosome" op="LOOKUP" value="2R" extraValue="D. melanogaster"></constraint></query>

The query returns a sequence-like object in JSON format.


```
{
   "features":[
      {
         "start":5866824,
         "seq":"CACTCGAGCTGTGACCGCCGCACAGTCAACAACTAACTGCCTTC
 ...
GCGCAAAATCAAATTAAGAAATAAATGCGAAAATAACATTG",
         "end":5868300
      }
   ],
   "executionTime":"2014.01.23 14:22::19",
   "wasSuccessful":true,
   "error":null,
   "statusCode":200
}
```


In most cases we would recommend that users unfamiliar with making raw HTTP requests take advantage of the automatic code generation available through the InterMine web interface.

c. Building analysis pipelines

Different web service resources and their methods can be joined to produce automated analysis pipelines. With basic programming skills it is possible to join fragments of InterMine's generated code to generate powerful analysis pipelines.

An example script to find similar genes which share a gene ontology (GO) annotation is shown below. The script calls a list from an InterMine instance and uses the list enrichment method (in this case GO) to analyse for genes enriched for GO terms. For the GO term with highest enrichment (if any), a query retrieves all genes (from the same organism) annotated with that GO term. A final step is to identify (*) which of the genes were already present in the starting list. By editing the URI and LIST parameters, the code can be used to suggest genes by enriched GO terms for any InterMine instance which loads gene GO annotations.

```python
from intermine.webservice import Service

URI = "http://www.mousemine.org/mousemine"
LIST = "Mouse DNA repair genes 2013-01-06"

def suggest_genes_by_go(uri, list_name, enrichment = "go_enrichment"):
    # Get a connection to the appropriate webservice, and get a representation of the
list.
    service = Service(URI)
    gene_list = service.get_list(list_name)

    # Check that the enrichment tool exists, and that the list name is correct.
    if enrichment not in service.widgets:
        raise Exception(enrichment + " is not one of the available enrichment tools: " +
str(set(w['name'] for w in service.widgets.values())))
    if gene_list is None:
        raise Exception(list_name + " not found")

    # Announce what we are doing.
    print "# Finding suggestions for", list_name, "from", uri, "using", enrichment

    # Find out what genes are in the list, and from which organisms.
    # We only want to suggest genes from the same organisms.
    organisms = service.model.Gene.\
                        select("symbol", "organism.taxonId").\
                        where("Gene", "IN", list_name)
    taxa = set()
    symbols = set()

    for row in organisms.rows():
        symbols.add(row["symbol"])
        taxa.add(row["organism.taxonId"])

    # Find out what the most characteristic GO term is for this list.
    most_enriched = next(gene_list.calculate_enrichment(enrichment))

    if most_enriched is None:
        raise Exception("No enriched item found")

    print "# Most enriched GO term:", most_enriched.description, most_enriched.identifier

    # Prepare a query that will find other genes in the appropriate organisms that
    # can be characterised by the same GO term.
    q = service.model.Gene.\
```

```
                where("organism.taxonId", "ONE OF", map(str, taxa)).\
                where("ontologyAnnotations.ontologyTerm.identifier", "=",
most_enriched.identifier)


    # Find and report the results.
    print "# Genes matching GO term: (* = already in list)"

    for gene in q.results():
        star = "*" if gene.symbol in symbols else ""
        print gene.symbol, gene.primaryIdentifier, star

# If run as a program, run against mousemine with the given list.
if __name__ == "__main__":
    suggest_genes_by_go(URI, LIST, "go_enrichment_for_feature")
```

Output:
Finding suggestions for `Mouse DNA repair genes 2013-01-06` from
`http://www.mousemine.org/mousemine` using `go_enrichment_for_feature`
Most enriched GO term: DNA metabolic process GO:0006259
Genes matching GO term: (* = already in list)
Pttg1 MGI:1353578 *
Mms19 MGI:1919449 *
Dntt MGI:98659
Rad51 MGI:97890 *
Parp1 MGI:1340806 *
Trex1 MGI:1328317 *
Tefm MGI:1915800
… more results ...

The Example Python code below joins InterMine's web services to create a simple translational biology pipeline. Using web services to query MouseMine, the code links list service, enrichment analysis and template search methods to retrieve human genes which are annotated with the same disease terms as the most enriched term for a set of mouse genes.

```
from intermine.webservice import Service

URI = "http://www.mousemine.org/mousemine"
LIST = "Mouse DNA repair genes 2013-01-06"
TOOL = "medic_enrichment_for_feature"
TEMPL = "HDisease_HGene"

# Instantiate a connection to a service, and get a
# representation of the list.
mousemine = Service(URI)
```

```
gene_list = mousemine.get_list(LIST)

# Find the most enriched term, in this case a disease term.
most_enriched = next(gene_list.calculate_enrichment(TOOL))

# Define the values we want to run the template with
param = {
    "op": "LOOKUP",
    "value": most_enriched.identifier,
    "extra_value": None
}
# Get a reference to the template by name.
genes_for_disease = mousemine.get_template(TEMPL)

print "Human genes matching", most_enriched.description

# Run the template, and print out the genes, with each of their GO terms.
for gene in genes_for_disease.results(A = param):
    terms = [oa.ontologyTerm.name for oa in gene.ontologyAnnotations]
    print gene.primaryIdentifier, gene.symbol, ";".join(terms)
```

Output:
Human genes matching DNA Repair-Deficiency Disorders
54840 APTX Ataxia, Early-Onset, with Oculomotor Apraxia and Hypoalbuminemia
472 ATM Ataxia-Telangiectasia
4361 MRE11A Ataxia-Telangiectasia-Like Disorder
6892 TAPBP Bare Lymphocyte Syndrome, Type I
6890 TAP1 Bare Lymphocyte Syndrome, Type I
6891 TAP2 Bare Lymphocyte Syndrome, Type I
4261 CIITA Bare Lymphocyte Syndrome, Type II
8625 RFXANK Bare Lymphocyte Syndrome, Type II
5993 RFX5 Bare Lymphocyte Syndrome, Type II
5994 RFXAP Bare Lymphocyte Syndrome, Type II
… more results ...
